# Construction of a Fluorescent H_2_O_2_ Biosensor with Chitosan 6-OH Immobilized β-Cyclodextrin Derivatives

**DOI:** 10.3390/md15090284

**Published:** 2017-09-04

**Authors:** Wenbo Dong, Weiping Li, Yu Chen, Yanchun Ye, Shaohua Jin

**Affiliations:** 1 School of Materials Science and Engineering, Beijing Institute of Technology, Beijing 100081, China; dwb194413@126.com (W.D.); jinshaohua@bit.edu.cn (S.J.); 2 School of Chemistry and Chemical Engineering, Beijing Institute of Technology, Beijing 100081, China; bitmsejournal@yeah.net (W.L.); ye_yanchun@sina.com (Y.Y.)

**Keywords:** chitosan, catalase, β-cyclodextrin, Rhodamine B, fluorescent biosensor

## Abstract

In the present work, a fluorescent H_2_O_2_ biosensor was constructed by encapsulating fluorescent probe Rhodamine B (RhmB) in the hydrophobic cavity of the cyclodextrin (β-CD) and immobilizing catalase (CAT) on the 2-NH_2_ of chitosan (CTS) in a chitosan 6-OH immobilized β-cyclodextrin derivative (CTS-6-CD). The inclusion complex of CTS-6-CD to RhmB (CTS-6-CD-RhmB) was prepared by a solution method. Its structure and inclusion efficiency were determined by Fourier transform infrared spectroscopy (FTIR), X-ray diffraction (XRD), and fluorescence spectroscopy (FL). CAT was immobilized on CTS-6-CD-RhmB to eventually form the functional membrane, CTS-6-CD-RhmB-CAT, via glutaraldehyde crosslinking, which was further characterized by FTIR and FL, and used as a H_2_O_2_ biosensor. The functional membrane was used to simultaneously oxidize and detect H_2_O_2_. The detection condition was optimized as pH 8, a reaction temperature of 25 °C, and an immobilized enzyme concentration of 2 × 10^−4^ mol/L. The fluorescence response of the biosensor exhibited a good linear relationship with the concentration of H_2_O_2_ in the range of 20 mΜ–300 μM and the detection limit of 10^−8^ mol/L.

## 1. Introduction

Reactive oxygen species (ROS) are involved in the initiation of biological effects of various factors. Their concentration can significantly affect many biological activities [1,2,3]. H_2_O_2_ is a representative of ROS. A low H_2_O_2_ concentration can be used as a signal transduction and expansion of the second messenger. High concentrations of H_2_O_2_ can cause oxidative stress and trigger a variety of diseases and physiological disorders [4,5]. Therefore, the real-time detection of H_2_O_2_ is highly desired to understand its relationship with human diseases, accurately diagnose diseases, and monitor disease development [6].

Various H_2_O_2_ detection techniques, such as spectrophotometry, high performance liquid chromatography, chemiluminescence, fluorescence spectrophotometry, electrochemical analysis, and so on, have been reported [7,8]. Among them, fluorescent biosensor has been used in a variety of fields, especially on-site H_2_O_2_ detection, due to its low cost, convenience, and high sensitivity. Recently, fluorescent H_2_O_2_ biosensors have attracted considerable attention. For example, Han [9] constructed three novel low cost and highly selective fluorescent H_2_O_2_ biosensors with silver nanoclusters and nucleic acid dyes. The biosensors were successfully used for the H_2_O_2_ detection in the presence of Fe^2+^ without any complex modification required.

Chitosan (CTS) is the only natural alkaline polysaccharide which possesses a unique 2-NH_2_ structure. Its excellent film-forming property, degradability, and biocompatibility make it an important carrier of biosensing films [10,11,12,13]. The modified CTS can significantly improve the detection performance of various biosensors.

β-cyclodextrin (β-CD) is hydrophilic due to its external hydroxyl groups, but possesses a hydrophobic cavity with a certain size [14]. Some small molecules can be included in the cavity of β-CD or its derivatives by hydrophobic, hydrogen, and van der Waals forces to form fluorescent biosensors. The force to include the small molecules decides the selectivity of the biosensors. In addition, β-CD can improve the solubility and stability of the included compounds [15], which thus increases the fluorescence intensity of the guest molecules, and significantly improves the sensitivity of the corresponding fluorescent sensors. Thus, β-CD has been widely used in fluorescence enhancement [16,17].

**Figure 1:**
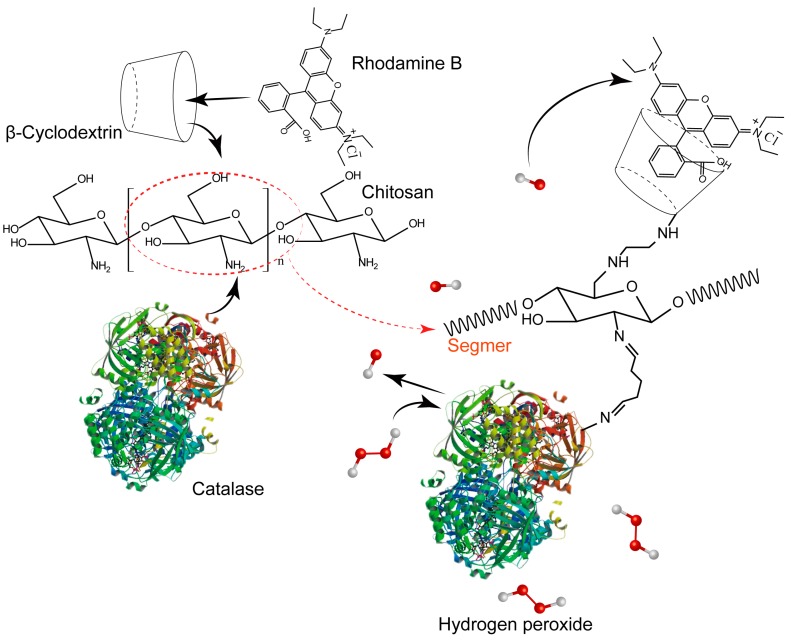
Schematic diagram of experimental structure and detection process.

To improve the performance of fluorescent biosensors, attempts have been made to combine the excellent properties of CTS with those of β-CD by modifying CTS with β-CD in biosensor construction. However, most studies have been focused on preparation of CTS-CD by the reaction of cyclodextrin derivatives with the highly active 2-NH_2_ groups on the chain of chitosan, which is unconducive to the utilization of the biologically active 2-NH_2_ of CTS. We previously systematically explored the preparation of various CTS-6-CD derivatives. For example, Chen et al. [18] immobilized cyclodextrin on the 6-OH of CTS by the nucleophilic substitution method, where the amino group of CTS was pre-protected with phthalic anhydride. The 2-NH_2_ was then deprotected with hydrazine hydrate. The resultant CTS-6-CD derivative exhibited good solubility with the biological activity of 2-NH_2_ unaffected. In the present work, we proposed a novel method for the construction of fluorescent H_2_O_2_ biosensors with CTS and β-CD. A catalase was immobilized on the 2-NH_2_ of CTS and the fluorescent probe rhodamine B (RhmB) was encapsulated in the hydrophobic cavity of the cyclodextrin immobilized on the 6-OH of CTS to form a functional membrane CTS-6-CD-RhmB-CAT (Figure 1). It was then used for the H_2_O_2_ detection due to the synergistic effect of these two functional groups. Such strategy was able to simplify the detection of H_2_O_2_ and the separation of catalase, and increase the stability of RhmB in detection. The oxidation and detection of H_2_O_2_ were realized on the same functional membrane.

## 2. Results and Discussion

### 2.1. Characterization of CTS-6-CD-RhmB

**Figure 2:**
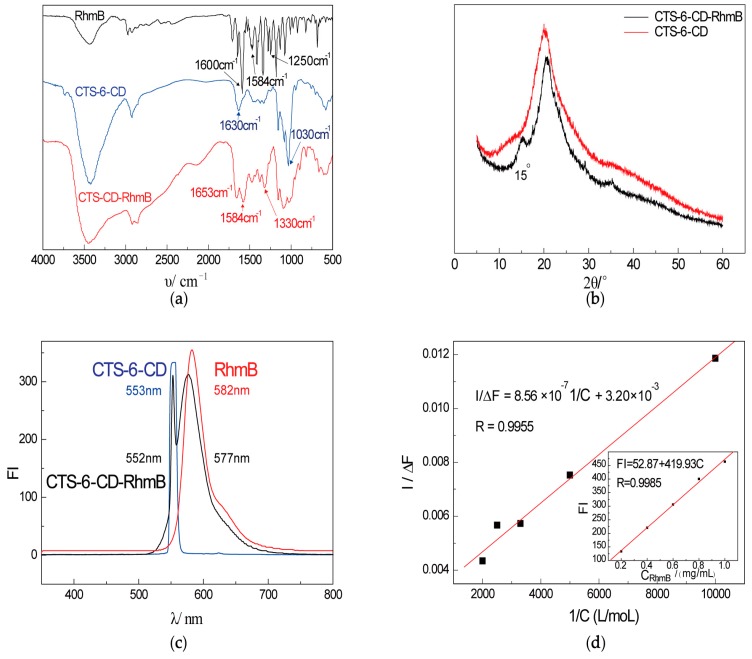
Characterization of CTS-6-CD-RhmB (**a**) Infrared spectrum of CTS-6-CD-RhmB, CTS-6-CD and RhmB; (**b**) XRD spectra of CTS-CD-RhmB and CTS-6-CD; (**c**) Fluorescence spectroscopy of CTS-6-CD-RhmB; (**d**) I/ΔF vs. 1/C curve and standard curve of inclusion effect of cyclodextrin on RhmB (the inserting figure).

The IR, XRD, and fluorescence emission spectra of CTS-6-CD-RhmB, CTS-6-CD, and RhmB are shown in Figure 2. It is clear that the IR spectrum of CTS-6-CD-RhmB is not a superposition of those of CTS-6-CD and RhmB (Figure 2a). The benzene ring vibration peaks of RhmB at 1600 cm^−1^ and 1480 cm^−1^ disappeared and the intensity of C–C stretching vibration peak at 1250 cm^−1^ was significantly reduced after the inclusion, indicating that the hydrophobic O-benzoic acid group of RhmB was included in the cavity of β-CD [19]. The intensities of the absorption peaks at 1030 cm^−1^ were attributed to the pyranyl groups of β-CD and CTS, respectively, were sharply decreased, and became wider after the inclusion due to the “inclusion compound infrared absorption attenuates effect” [20]. The strong and broad band at 3400 cm^−1^ that was ascribed to the hydrogen bond between the hydroxyl group and amine group of CTS-6-CD was weakened after the inclusion due to the increased distance between CTS chain by the included RhmB. For the XRD spectrum, the inclusion of RhmB in CTS-6-CD led to a new extra diffraction peak at 15°, and two weak peaks at ~28° and 35°. By the way, the intensity of CTS-6-CD around 2θ = 20° was decreased (Figure 2b). It can be explained that RhmB was included in CTS-6-CD via hydrogen bonding and van der Waals force, which increased the irregularity of the macromolecular chain and weakened the intensities of its diffraction signals, and the peaks assigned to the structure of RhmB stretched outside the cavity of cyclodextrin emerged. The fluorescence spectra of RhmB, CTS-6-CD, and CTS-6-CD-RhmB were compared. It could be found that RhmB exhibited a strong emission peak at 582 nm and CTS-6-CD exhibited no emission peak under the excitation at 552 nm (Figure 2c). The inclusion of RhmB in CTS-6-CD caused a blueshift of its emission peak because the inclusion limited the movement of the rhodamine molecules and thus caused certain damages to the conjugate system. These results indicate that RhmB was successfully included in CTS-6-CD.

The standard curve of the cyclodextrin clathrate to RhmB is shown in the main curve of Figure 2d. 1/(F − F_0_) exhibited a linear relationship with 1/C_0_, indicating the inclusion ratio was 1:1. The inclusion constant K was calculated to be 3.74 × 10^3^, indicating that the inclusion reaction occurred spontaneously at room temperature.

The inclusion efficiency was calculated to be 44.6% based on the load capacity of CTS-6-CD of 212.16 μmol/g and the inclusion amount of 94.57 μmol/g. Since cyclodextrin was immobilized on the CTS molecular chain, the degree of freedom of its molecular movement was limited. In addition, it tends to curl in solvents, and is thus partially shielded. Therefore, the inclusion efficiency of CTS-6-CD was lower than that of free β-CD, but still reached ~50%. It was calculated from the standard curve of inclusion effect in the inserting figure of Figure 2d according to the formula in Section 3.5 that the maximum inclusion amount of RhmB in free β-CD is ~150 μmol/g. The inclusion amount of RhmB in the immobilized β-CD reached 63% of the maximum inclusion amount of RhmB in free β-CD, indicating that the inclusion complex was able to maintain the sensitivity and accuracy of the β-CD based sensor.

CTS-6-CD has excellent film-forming properties due to its CTS macromolecule chains, which can increase the stability of its modified electrode. The 2-NH_2_ group on the CTS backbone in CTS-6-CD provides a binding site for multiple enzymes. The hydrophobic cavity of the cyclodextrin in CTS-6-CD provides a suitable environment for the inclusion of many small molecules. The immobilization promotes chemical stability. Therefore, CTS-6-CD is an excellent material for formation of single or composite functional membranes.

### 2.2. Characterization of CTS-6-CD-RhmB-CAT

**Figure 3:**
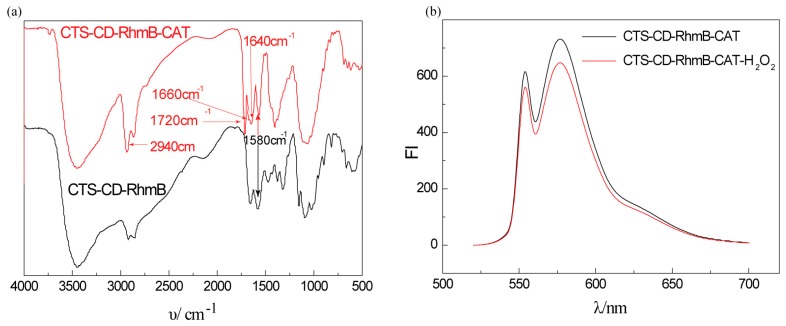
Characterization of CTS-6-CD-RhmB-CAT (**a**) Infrared spectrum of CTS-6-CD-RhmB-CAT and CTS-6-CD-RhmB; (**b**) The influence of the addition of H_2_O_2_ on fluorescence spectroscopy of CTS-6-CD-RhmB-CAT.

The immobilization of CAT on CTS-CD-RhmB weaken the IR absorption peak of the rocking vibration of -NH_2_ at 1580 cm^−1^, but significantly enhanced the stretching vibration absorption peak of C=N at 1640 cm^−1^ (Figure 3a). It can be explained that the 2-NH_2_ in CTS was occupied by glutaraldehyde to form a Schiff base [21,22]. The absorption peak at 1720 cm^−1^ was attributed to the suspended aldehyde group that was not linked with the enzyme [23]. A new peak appeared in the amide I band at 1660 cm^−1^ due to the stretching vibration of carbonyl in the skeleton peptide chain [24]. As shown in Figure 3b, the addition of H_2_O_2_ significantly reduced the fluorescence intensity of CTS-6-CD-RhmB-CAT due to the static fluorescence quenching where H_2_O_2_ was decomposed by CAT into hydroxyl radicals, and the included RhmB reacted with the hydroxyl radicals to form a non-fluorescent substance. These results indicate that CTS-6-CD-RhmB-CAT was successfully prepared.

### 2.3. Fluorescence Detection of H_2_O_2_ by CTS-6-CD-RhmB-CAT

The condition for the fluorescence detection of H_2_O_2_ by CTS-6-CD-RhmB-CAT was optimized to evaluate the detection performance.

**Figure 4:**
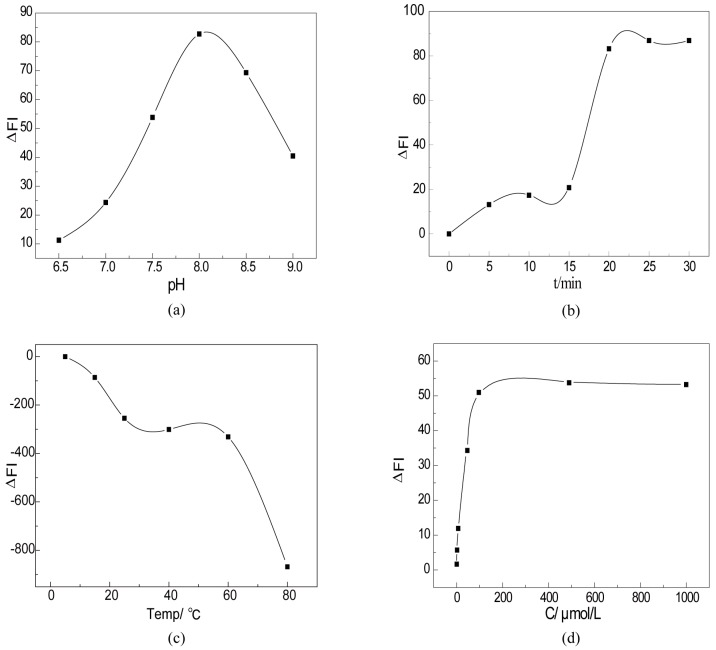
Optimization of H_2_O_2_ detection conditions (**a**) The difference of fluorescence intensity (ΔFI) with the change of pH; (**b**) ΔFI with the change of TIME; (**c**) ΔFI with the change of TEMP; (**d**) ΔFI with the change of enzyme concentration.

Figure 4a shows the effects of pH on the fluorescent response of CTS-6-CD-RhmB-CAT to H_2_O_2_. It is clear that CTS-6-CD-RhmB-CAT exhibited the strongest response to H_2_O_2_ at pH ~8. It can be explained that H_2_O_2_ was decomposed into hydroxyl radicals under the catalysis of catalase. The hydroxyl radicals oxidized RhmB and quench its fluorescence. Catalases have the highest activity under the biological condition. The catalase produced the highest amount of hydroxyl radical at pH ~8. Therefore, the pH for the H_2_O_2_ detection by CTS-6-CD-RhmB-CAT was optimized as 8.

The effects of reaction time on the fluorescence quenching of CTS-6-CD-RhmB-CAT by H_2_O_2_ were also investigated. It can be seen from Figure 4b that the fluorescence intensity remained stable within 10 min, significantly reduced at 15 min, and remained stable after 25 min reaction. The CTS-6-CD-RhmB-CAT exhibited certain fluorescence even after the reaction, indicating that the reaction between H_2_O_2_ and the enzyme was complete. Therefore, the time for the reaction between CTS-6-CD-RhmB-CAT and H_2_O_2_ was optimized to be 25 min. Su et al. [25] successfully synthesized a fluorescent gold nanoclusters and stabilized it with lysozyme under alkaline conditions for the diction of alkaline protease. They found that the optimal reaction time for the reaction was 3 h. In contrast, the biosensors we constructed in the present work significantly shortened the detection time and realized rapid detection for H_2_O_2_.

Figure 4c shows the effects of temperature on the H_2_O_2_ detection. The fluorescence quenching effect dramatically increased with the increase of temperature from 0 °C to 25 °C. It can be explained that the collision frequency between the catalase immobilized on the chitosan derivative and H_2_O_2_ was low at low temperatures, e.g., the collision between fluorescent substance and target molecules was low, resulting in weak fluorescence quenching effect. The collision frequency between the immobilized catalase and H_2_O_2_ increased with the increase of temperature, resulting in stronger fluorescence quenching effects. As the temperature further increased to 60 °C, no significant change was found in the fluorescence intensity. In the temperature range of 25–60 °C, the fluorescence quenching effect increases with the increase of temperature. Meanwhile, the fluorescence emission efficiency and fluorescence intensity of CTS-6-CD-RhmB-CAT in the solution decrease with the increase of temperature due to the decomposition of RhmB at high temperatures. In addition, optimum active temperature of the catalase is 25 °C. High temperatures can deactivate the enzyme, leading to a decreased capacity to produce hydroxyl radicals. Therefore, the detection temperature was optimized at 25 °C.

CTS-6-CD-RhmB-CAT concentration can directly affect the sensitivity and resolution of the biosensor. A low concentration leads to low sensitivity towards the target substances. Extremely high concentrations reduce the efficiency of the sensor and utilization of the derivative. Therefore, the optimization of CTS-6-CD-RhmB-CAT concentration is of paramount importance. Figure 4d shows the effects of CTS-6-CD-RhmB-CAT concentration on H_2_O_2_ detection. The fluorescence quenching effect linearly increased with the increase of CTS-6-CD-RhmB-CAT concentration from 0.1 to 200 μmol/L. Further increasing CTS-6-CD-RhmB-CATconcentration showed no significant effect on the fluorescence quenching effect, indicating increasing the concentration of CTS-6-CD-RhmB-CAT to over 200 μmol/L had no significant influence on the detection sensitivity. Therefore, the optimal CTS-6-CD-RhmB-CAT concentration was determined to be 200 μmol/L.

Based on these results, the optimal condition for the detection of H_2_O_2_ by the CTS-6-CD-RhmB-CAT biosensor can be summarized as follows: pH 8.0, the reaction temperature of 25 °C, and the CTS-6-CD-RhmB-CAT concentration of 200 μmol/L.

### 2.4. Quantitative Detection of H_2_O_2_

**Figure 5:**
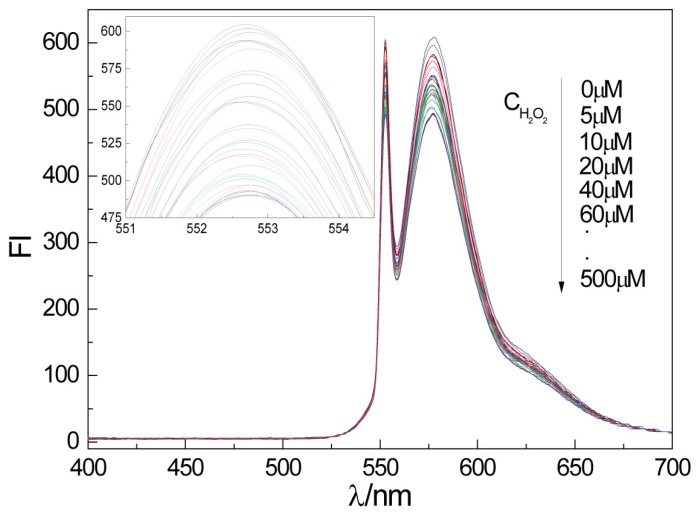
Fluorescence response of CTS-6-CD-RhmB-CAT to different concentrations of H_2_O_2_.

The fluorescence responses of CTS-6-CD-RhmB-CAT biosensor to different concentrations of H_2_O_2_ under the optimal condition are shown in Figure 5.

**Figure 6:**
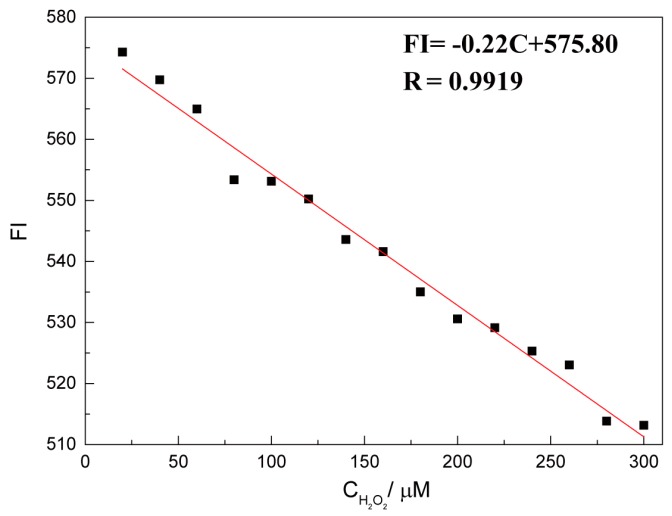
Linear relationship between fluorescence intensity and H_2_O_2_ concentration.

It is clear that the fluorescence intensity of CTS-6-CD-RhmB-CAT was gradually decreased with the increase of H_2_O_2_ concentration (Figure 5). CTS-6-CD-RhmB-CAT exhibited a strong fluorescence emission at 552 nm that varied with the change of H_2_O_2_ concentration. To more intuitively determine the relationship between fluorescence intensity (FI) and H_2_O_2_ concentration (C), the fluorescence intensity at 552 nm was plotted vs. H_2_O_2_ concentration (Figure 6). The result indicates that the fluorescence intensity of the CTS-6-CD-RhmB-CAT biosensor has an excellent linear relationship with H_2_O_2_ concentration in the range of 20 mΜ–300 μM with the fitting equation

FI = −0.22C + 575.80 (R = 0.9919)

Based on the linear regression curve, the detection limit of the H_2_O_2_ biosensor was determined to be 10^−8^ mol/L. Jiang et al. prepared an electrochemical H_2_O_2_ sensor with chitosan, carbon nanotubes, and immobilized catalase. The sensor exhibited a detection limit of 2.5 μmol and a linear detection range of 5–50 μM. Sedigheh et al. fabricated an electrochemical H_2_O_2_ sensor with a detection limit of 8.7 μmol and a linear detection range of 10–100 μmol by immobilizing catalase on a multi-walled nanotube and thionine membrane [26]. Compared with these H_2_O_2_ sensors, our CTS-6-CD-RhmB-CAT functional membrane biosensor has a simple structure, but with a lower detection limit and much wider detection range.

## 3. Materials and Methods

### 3.1. Materials

Chemical grade rhodamine B (RhmB) was purchased from Tianjin Shibo Chemical Co., Ltd. (Tianjin, China). β-Cyclodextrin (A.R.) was supplied by Tianjin kwangfu Fine Chemical Industry Research Institute (Tianjin, China). Chitosan (CTS) with a deacetylation degree of 95% and molecular weight of 1.1 × 10^6^ was provided by Zhejiang Aoxing Biochemical Co., Ltd. (Yuhuan, Zhejiang, China). Catalase (3500 units/mg) was purchased from Aladdin Industrial Co., Ltd. (Shanghai, China). The chitosan 6-OH-loaded cyclodextrin derivative (CTS-6-CD) was prepared as described previously [18]. The loading of cyclodextrin was determined to be 212.16 μmol/g. Glutaraldehyde was purchased from Tianjin Fuchen Chemical Co., Ltd. (Tianjin, China). Other reagents were all analytical grade and used as received.

### 3.2. Synthesis of CTS-6-CD-RhmB

CTS-6-CD was dissolved in ethylenediamine-ethanol (v:v = 1:1) solution. Excessive RhmB was added to the CTS-6-CD solution and refluxed in an oil bath at 60 °C for 6 h. The produced precipitate was washed with secondary deionized water and ethylene glycol until the wash solution became neutral, and low-temperature vacuum dried to afford CTS-6-CD-RhmB inclusion compound.

### 3.3. Synthesis of CTS-6-CD-RhmB-CAT

The obtained CTS-6-CD-RhmB was dissolved in 1% acetic acid aqueous solution at m_CTS-6-CD-RhmB_/m_Acetic acid_ = 3%, allowed to swell for 3 h, and neutralized with a sodium hydroxide solution. Glutaraldehyde was then added to the neutralized CTS-6-CD-RhmB solution with a mass fraction of 2.5%, allowed to react for 5 min, mixed with a 2.5% (*w*/*v*) catalase (CAT) solution, and allowed to react at 4 °C for 120 min. The product was vacuum filtered and washed with distilled water and phosphate buffer to afford CTS-6-CD-RhmB-CAT.

### 3.4. Characterization of CTS-6-CD-RhmB and CTS-6-CD-RhmB-CAT

The IR spectra of CTS-6-CD-RhmB and CTS-6-CD-RhmB-CAT was recorded on a NicoLet NEXUS-470 Fourier transform infrared spectroscopy using KBr pellets.

The XRD patterns of CTS-6-CD and CTS-6-CD-RhmB were collected on an ALC-100.4 polycrystalline X-ray diffractometer (Beijing Sartorius Instrument Systems Co., Ltd., Beijing, China) in the θ range of 0°–60°.

The fluorescent emission spectra of CTS-6-CD-RhmB-CAT and CTS-6-CD-RhmB were measured under the excitation at 552 nm with both excitation and the emission slit widths set to 2 nm using a 970CRT type fluorescence spectrophotometer (Shanghai Precision Science Instrument Co., Ltd., Shanghai, China).

### 3.5. Determination of Inclusion Constant, Inclusion Amount, and Inclusion Efficiency

The reaction between β-CD and RhmB can be expressed as

CD + *n*RhmB = [RhmB]*_n_* − CD

The equilibrium constant can be calculated as

K = [C_β-CD-RhmB_]·[CD]^−1^·[RhmB]^−n^

If the inclusion ratio is 1:1, then

1/(F − F_0_) = 1/(αC_RhmB0_C_β0_K) + 1/(αC_RhmB0_)

where C_RhmB0_ is the initial concentration of RhmB (10^−5^ mol/L), C_β0_ is the initial concentration of β-CD (10^−4^ mol/L), and F − F_0_ is the difference between the absorptions before and after β-CD added.

To calculate the inclusion constant, K, β-CD/RhmB dimethyl sulfoxide solutions with different mole ratios were prepared and their absorptions were measured in the range of 300–700 nm with a TU-1810 UV–Vis spectrophotometer (General Analytical Instrument Co., Ltd., Beijing, China) to establish a fitting curve of 1/(F − F_0_) vs. β-CD concentration. The β-CD concentration of 0 mol/L was used as the initial condition.

To determine the inclusion amount, RhmB solutions of different concentrations in diluted sulfuric acid were prepared, and measured for their fluorescent emission to establish a standard curve equation of

F = 419.93CRhmB + 52.87 (R^2^ = 0.9985)

The concentration of RhmB in the inclusion solution was obtained by fitting the standard curve equation. The inclusion amount was then calculated using

I = CV/Mm
(1)

where I is the amount of guest molecules per gram of the inclusion product (μmoL/g), C is the concentration of RhmB obtained from the standard curve equation (mg/mL), V is the volume of the inclusion compound solution, M is the molecular weight of the guest molecule, and m is the mass of clathrate compound.

## 4. Conclusions

Based on the excellent properties of chitosan, the high bioactivity of 2-NH_2_ on the skeleton of chitosan, and the hydrophobic cavity of cyclodextrin, a functional membrane CTS-CD-RhmB-CAT was prepared and its application in rapid H_2_O_2_ detection was explored. RhmB was included in CTS-6-CD derivative by a solution method. The clathrate product with glutaraldehyde was further linked with CAT to form a fluorescent biosensor for quantitative detection of H_2_O_2_. The detection condition was optimized as pH 8, the reaction temperature of 25 °C, and the CTS-6-CD-RhmB-CAT concentration of 200 μmol/L. The fluorescence response of the fluorescent biosensor exhibited a good linear relationship with H_2_O_2_ concentration in the range of 20 μM–300 μM and the detection limit is 10^−8^ mol/L, indicating the high sensitivity of the biosensor. Our work provides a new approach to the application of the important chitosan derivative, CTS-6-CD.
